# Referral hospitals in the Democratic Republic of Congo as complex adaptive systems: similar program, different dynamics

**DOI:** 10.11604/pamj.2015.20.281.4266

**Published:** 2015-03-23

**Authors:** Hermès Karemere, Nathalie Ribesse, Jean-Bosco Kahindo, Jean Macq

**Affiliations:** 1 Université Catholique de Bukavu, République Démocratique du Congo; 2 Université Catholique de Louvain, IRSS (Institut de Recherche Santé et Société), Belgique; 3 Cemubac (Centre scientifique et médical de l'Université libre de Bruxelles pour ses activités de coopération), République Démocratique du Congo

**Keywords:** Complex adaptive system, district hospitals, governance, aid development evaluation, Democratic Republic of Congo

## Abstract

**Introduction:**

In many African countries, first referral hospitals received little attention from development agencies until recently. We report the evolution of two of them in an unstable region like Eastern Democratic Republic of Congo when receiving the support from development aid program. Specifically, we aimed at studying how actors’ network and institutional framework evolved over time and what could matter the most when looking at their performance in such an environment.

**Methods:**

We performed two cases studies between 2006 and 2010. We used multiple sources of data: reports to document events; health information system for hospital services production, and “key-informants” interviews to interpret the relation between interventions and services production. Our analysis was inspired from complex adaptive system theory. It started from the analysis of events implementation, to explore interaction process between the main agents in each hospital, and the consequence it could have on hospital health services production. This led to the development of new theoretical propositions.

**Results:**

Two events implemented in the frame of the development aid program were identified by most of the key-informants interviewed as having the greatest impact on hospital performance: the development of a hospital plan and the performance based financing. They resulted in contrasting interaction process between the main agents between the two hospitals. Two groups of services production were reviewed: consultation at outpatient department and admissions, and surgery. The evolution of both groups of services production were different between both hospitals.

**Conclusion:**

By studying two first referral hospitals through the lens of a Complex Adaptive System, their performance in a context of development aid takes a different meaning. Success is not only measured through increased hospital production but through meaningful process of hospital agents’” network adaptation. Expected process is not necessarily a change; strengthened equilibrium and existing institutional arrangement may be a preferable result. Much more attention should be given in future international aid to the proper understanding of the hospital adaptation capacities.

## Introduction

In many African countries, development aid agencies focused during years on primary care or disease focused health programs. Until recently, district or first referral hospitals received little attention. It has been the case for the Democratic Republic of Congo (DRC) untill the health system re-building process started. Specifically, since 2006, the development of the strategy to strengthen the DRC healthcare system aimed at revitalizing the health zone or health district (HD) [1]. The first referral hospital (FRH) has been considered at the center of these changes [2]. It is in this context that the Health Program of the 9th European Development Fund (PS9FED) supported the development of health services in four DRC provinces [1], including the Eastern Province where both Bunia and Logo HD are located. These two HD were among those targeted by the PS9FED [3]. Although Paris declaration, Accra agenda, and more recent Busan meeting have promoted paradigm changes (i.e. strengthened local ownership, enhanced harmonization and alignment), aid effectiveness is still mostly evaluated through project or program predefined objectives [4]. This has been the case for PS9FED. Objectives of that program were to improve both the performance of health services and access to health care for the population.

We aimed in this study to take another perspective. We studied how governance influences the “adaptation” of two first referral hospitals (Bunia and Logo) to PS9FED program in an unstable environment such as Ituri in the Eastern DRC region [5–7]. Governance has been defined and interpreted in many ways. We adopted the approach of Rijke who described it in terms of (1) actor's network, (2) institutional framework (i.e. administrative and other working rules that structures the actors’ interactions) and (3) processes (i.e. leadership and social learning that will influence over time actor's network and institutional framework) [8]. In order to study the hospitals governance, we used the lenses of Complex Adaptive Systems (CAS). A CAS is a collection of agents (i.e. first dimension of governance, the equivalent of actors’ network). Each of them has their own behavior, responsibilities and characteristics. The characteristic of one agent is influenced by the behavior of other agents, being part of their context. The interactions between agents influence the overall CAS behavior and can be summarized through simple rules (i.e. the second dimension of governance, the institutional framework) [9–12]. As a consequence of the interactions between agents, CAS do have a “behavior” that can be explained through some key characteristics. Two of them will be illustrated in this study: path dependency and transition phase. Path dependency reflect the fact that “non-reversible processes have similar starting points yet lead to different outcomes, even if they follow the same rules, and outcomes are sensitive not only to initial conditions, but also to bifurcations and choices made along the way” [13]. Transition phase is point in time or event when radical changes take place “in the features of system parameters as they reach certain critical points” [13]. Thus, the purpose of this study was therefore to study in two FRH, how two dimensions of governance (actors’ network and institutional framework) evolved over time and what could matter the most when looking at their performance, using the lenses of CAS, in a changing environment.

## Methods

We performed two cases studies of Bunia and Logo FRH between 2006 and 2010, by considering them as CAS. Specifically, we tried to identify key characteristics of agents’ interactions by interpreting the relation between evolution of hospital production and key events or activities performed in the frame of PS9FED. Thus, our approach was mainly inductive and our aim was to identify emerging theoretical propositions as a result of the case studies interpretation [14]. We chose case study as a methodology for its capacity to tackle issues under study with a perspective of complexity [15]. We chose to perform it over time (i.e. with a longitudinal dimension) to study governance as a process and to investigate adaptation process.

### Setting and selection of cases studies

Bunia and Logo Hospitals were chosen because they were considered to be amongst the best in the region before the PS9FED started [3]. Additionally, they both had a functional health information system. The Bunia FRH is located in the town of Bunia. It is part of the HD of Bunia. In 2010, this HD had an estimated population of 239,863 inhabitants, of which 25% lived in rural areas. The HD covered an area of 450 square km2, had 16 health centers, 7 private hospitals, 1 FRH and 31 health posts. The main cause of death in the HD was malaria. Moreover, this HD also recorded epidemics including cholera, which affected 371 cases and caused 5 deaths in 2007. The Logo FRH is part of Logo HD. In 2010, this HD had an estimated population of 208,716 inhabitants. It has 20 health centres, a FRH and 22 private hospitals, relatively unregulated health-posts that are scattered throughout the district. Records show the existence of rare diseases like the plague (238 cases in 2007, and 4 cases in 2010). The main cause of death is malaria. Over the past few years, a few epidemics, namely meningitis, cholera and bloody diarrhea, have been recorded in the health district. Violence, including sexual violence, is also an emerging issue. Both Bunia and Logo districts were affected by the war between 1996 and 2003. There were looting and destruction of several health facilities. In Bunia town, economic activities were interrupted and infrastructure destroyed. In Logo, population was partially displaced but returned to their field between fighting. PS9FED implemented a similar set of interventions in Bunia and Logo from 2006 onwards. Health infrastructures were rehabilitated and equipped; financial incentives and in-services trainings were given to health personnel; stocks of drugs were restored; and services were subventionned for the population. These interventions were implemented in hospital and health centers. District health team also received support to do supervision and meetings, manage health information system, perform resources management, and epidemiologic surveillance. Both districts received in-situ technical assistance. More details of some of the intervention targeting both hospitals will be further developed in the result section.

### Data collection and analysis

The case study is facing a situation in which particular technical interest focuses more on variables than on one dataset. Data analysis in this case is based on multiple data sources that require convergence through a triangulation method. It benefits from prior development of theoretical propositions that have guided the data collection [16]. In this study, we used multiple sources and mixed data (quantitative-qualitative). First, we documented events or interventions that took place in the hospitals, associated or unassociated with the implementation of PS9FED, as well as with sudden changes observed in hospital production during the study period. The main sources used were the annual reports of the two hospitals, the reports from training workshops, reports from management or board of directors’ workshops, and reports from mission partners or intermediate executives (at the provincial level). This resulted in the determination of the main events and interventions that occurred in both hospitals during this time. This was triangulated with key-informants interviews. Secondly, we studied hospital services production. The data were prospectively collected for that purpose between 2006 and 2010 using the hospitals’ routine health information systems. The analysis looked at the evolution of the annual production of hospital care in the Bunia and Logo hospitals using eight different indicators: the number of new consultations, the number of hospital admissions, the number of major surgical interventions, the number of appendectomies, the number of blood transfusions, the number of ultrasounds, the number of births and the number of caesarean sections. This data was then graphically displayed in a line graph, with three curves representing: “consultations - hospital admissions,” “surgery,” and “maternity.” Representing the data in the form of curves facilitated interpretation as it allowed for a visual analysis of production trends; it was easy to identify sudden changes or stable periods. The reasons behind sudden changes were explored using the qualitative data. Thirdly, we interpreted the relation between interventions and production of services through “key-informants” interviews. A total of 24 interviews (Bunia, n = 12 and Logo, n = 12) were conducted with key informants, chosen deliberately on the basis of their level of involvement in each hospital's management. These informants included district medical officers, medical hospital director, medical head of staff, nursing directors, hospital management administrators, nursing supervisors, intermediate executives, and technical assistants working in non-governmental organizations supporting the hospital. The interviews were conducted from May to July 2010 for the Logo Hospital and from May to December 2010 for the Bunia hospital. The interviews used open-ended questions for each hospital. The questionnaire was based on the analysis of the evolution of hospital indicators and of events and interventions that took place in each hospital. It was administered directly to each informant, and included information about events and evolution of the services production. The interviews bore upon Bunia and Logo's hospitals’ internal and external actors. Each interview lasted an average of 60 minutes. We coded the actors’ responses by hospital KI 1(Key-Informant 1), KI2, KI3, and so on, in order to better analyze the interactions between agents.

**Figure 1:**
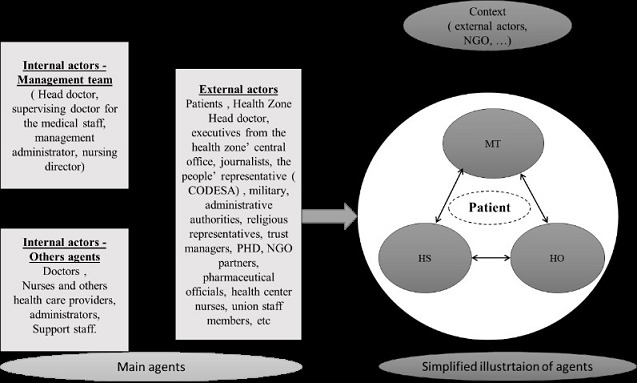
Agents and their interactions at the Logo and Bunia hospitals

We divided the main agents into internal agents and external agents. Internal agents include the management team, other staff members and hospital owners. The management team is made up of a head doctor, a supervising doctor for the medical staff, a nursing director, and a hospital management administrator. Other staff members are doctors, nurses and other healthcare providers, administrators and support staff. The basic profile of other staff members is the same for both hospitals, even if their skills differ for certain positions. Hospital owners may have a strong influence on the management team. They are either the diocese in Logo, or the ministry of health in the case of Bunia. External agents are mainly patients, the health district head doctor, other health district executives, managers of health insurance organizations, journalists, the community's representatives (in particular, the Health and Development Committee), the military, administrative authorities, representatives of religious organizations, trust managers, Provincial Health Division executives, international non-governmental organization technical assistants, pharmaceutical officials, health center nurses, and union staff members. External agents differ between the two hospitals. The community representatives have a strong influence in Logo; they contribute to the hospital's funding and participate in hospital actions. In Bunia, the staff union plays a major role in negotiating decisions that directly affect the staff. The international NGO technical assistants working in local HD and their hospitals have different methodological approaches; some rely heavily on the HD management team that they train, while others, in addition to relying on the HD management team, strengthen other hospital agents through a permanent support system. For clarification purposes, we simplified the reality of agent influences in Logo and Bunia. Because of the focus on hospital governance, and management, we considered three main internal agents in the face of change: (1) the management team (MT), (2) the hospital staff (HS) and (3) hospital owners (HO). Among external agents, we focused on technical assistants (TA) employed by the PS9FED. They are represented in Figure 1.

Interpretative analysis focused on the main agents in each hospital, allowed for analysis of the characteristics of their interactions, helped to understand actors’ points of views on hospital events and interventions, and, finally, helped uncover factors underlying the sudden changes observed in the hospital care production.

### Ethical considerations

This study was observational (i.e. observing a program implementation within two hospitals). Before its implementation, the research protocol was submitted to the authorities of the Ministry of Health of the Democratic Republic of Congo for approbation and it has been discussed by the GRAP-PA (*Groupe de recherche en appui à la politique sur la mise en œuvre de l'agenda pour l'efficacité de l'aide à la suite des déclarations de Paris et d'Accra*). It was also part of a doctoral thesis. In that frame, protocol has been reviewed by the Doctoral Committee of the Catholic University of Louvain. Participants provided their verbal informed consent to participate in this study. All participants in interview were actors in the program implementation and versed before in interviews related to the program over multiple evaluations performed. Written consent was thus deemed unnecessary. The Doctoral Committee approved procedure. This study didn't require any other ethical approval.

## Results

### Main events that occurred in the Bunia and Logo hospitals

**Figure 2:**
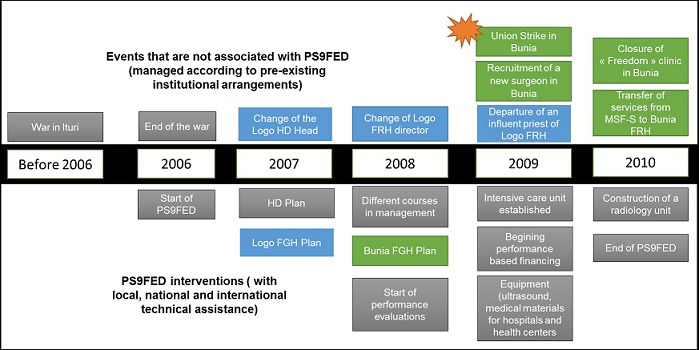
Main events and interventions between 2006 and 2010 in Bunia and in Logo

Twenty one interventions and events were identified and validated by the key-informants’ interviews. Events were grouped in 3 types: events and interventions that are identical in the two hospitals (mostly related to PS9FED interventions), events and interventions specific to the Bunia hospital, and those specific to the Logo hospital. Some events were related to the context (i.e. the war in Ituri, closure of other health facilities ‘ MSF or ‘Freedom’ hospital in Bunia). Others were interventions that aimed at improving hospital management and governance (i.e. hospital or health district development plan ‘ Hospital or HD plan; performance based financing; management training). Third type of events where interventions to strengthen clinical capacities of hospital (intensive care unit ‘ ICU- organization, echography, XRay services ‘). Finally, series of events were related to human resources functioning (strikes in Bunia, changes in key staff- DMO and hospital director in Logo, departure of charismatic leader, new surgeon’). All these events are presented in a timeline on Figure 2.

Amongst the events listed, two, related to PS9FED, were identified by most of the key-informants interviewed (23 over 24) as having the greatest impact on hospital performance. These two events are the development of a hospital plan and the performance based financing. We present hereafter, the perception of key-informants for these 2 activities.

### Concerning the hospital plan, it was developed in 2007 in Logo and in 2008 in Bunia

Hospital plan was developed in 3 steps: a diagnosis to identify difficulties of the hospital to respond to health care demand of the population; a dialogue between hospital stakeholders to reach a consensus on priorities; a redaction of action plans for each priority by an ad-hoc committee including hospital authorities and technical assistance. The redaction had-hoc committee used slightly different methodology in Logo and Bunia, but, in both places, plans were short term (between a trimester and 100 days). Each hospital plan resulted in medical, nursing, managerial/administrative, capacity building and additional projects. Finally, an equipment, a training, a social and a master plan were derived from those projects. A technical monitoring and evaluation committee was appointed and plans were formally approved by the hospital management committee. In both hospitals, this was perceived as a new exercise but the design process was different.

### Concerning performance based financing (PBF); it started in both hospitals in 2009

**Figure 3:**
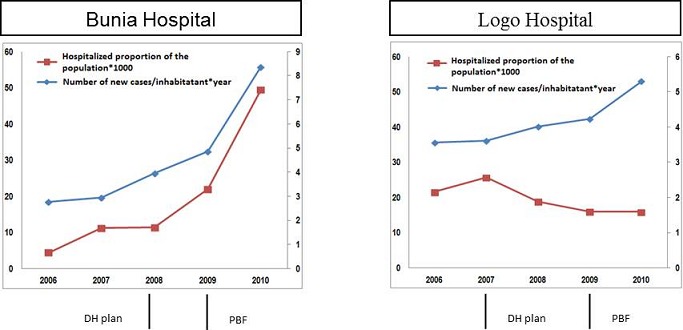
Evolution of consultations and admissions for the two hospitals

The form of PBF, introduced by PS9FED, included the creation of the Establishment of the Public Utility Fund for the Purchase of Health Services (EUP FASS). PBF was implemented progressively. From 2006 onwards, a « simple PBF » was introduced with payment in the form of drugs supply. From 2007, a payment in-cash was added to drugs supply. The establishment of the Public Utility Fund for the Purchase of Health Services (EUP FASS) started in 2009, and finally, peer evaluation of the quality began in 2010. Only certified structures (fulfilling basic quality criteria such as agreement from ministry of health, sufficient utilization rate, capacity to provide minimum package of activity, etc.) participated to PBF. Two types of contracts were implemented: the « integration » contract financing only curative consultations, providing subsidies in the form drugs; and the “progression” contract for the structures fulfilling, at the best, basic criteria. This contract financed a larger package of services (curative and preventive consultations, referrals, other hospital services...). Additionally, a quality bonus was progressively integrated in the scheme from 2010. This was based on peer evaluation. The role of EUP FASS was to pay the structures. The PBF reported in this article concerned “progression contract” in which both hospitals. Quality bonus was implemented after the reported period. In both hospitals, it was perceived as a powerful intervention to increase financial accessibility to the population. Production trends in hospital care between 2006 and 2010, and interpretations of interviewed agents on these trends. Two groups of services production were reviewed: (1) consultation at outpatient department (OPD) and admissions, and (2) surgery, including cesarean sections and blood transfusion services. Services within a group were expected to influence each other. The first group includes OPD consultations (number of new cases per inhabitants and per year) and hospital admissions (proportion of the population hospitalized). Initially (2006), the OPD consultations were lower at the Bunia hospital than at the Logo hospital. It increased thereafter in both hospital, but more sharply for Bunia than in Logo. Attendance ended-up to be higher in Bunia than in Logo. Concerning proportion of hospitalized person among the population, it was initially lower at the Bunia hospital than at the Logo hospital. Thereafter, it steadily increased each year until it far exceeded the Logo hospital in 2010. For Logo hospital, proportion began to slowly decline between 2007 and 2008, and then stabilized between 2008 and 2010. Key-informant interpretation of this evolution was that, in Bunia, the increase in consultations and hospital admissions was related both to PS9FED activities, including the PBFstarting in 2009, and to the closure of the MSF-Swiss hospital. In Logo, the increase in consultations was related to various PS9FED activities, and much more to the PBF (including subventions for the patients). The decrease in the number of hospitalizations was interpreted by the key informants as the result of an improvement in the quality of primary care throughout all health centers in the HD, mainly supported by PS9FED. This interpretation will be discussed further (Figure 3).

**Figure 4:**
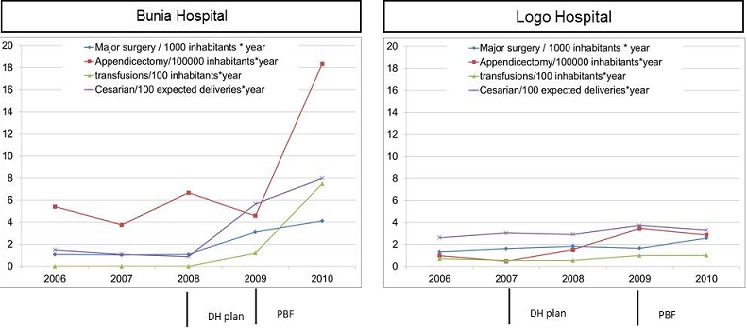
Trends in surgical acts in both hospitals

The second group included surgical activities and blood transfusion. Between 2006 and 2010, the number of major interventions, as well as the number of transfusions, significantly increased at the Bunia hospital; these numbers remained almost stationary at the Logo hospital. Looking a bit more precisely at the evolution of particular surgical intervention in Bunia, the rate of appendectomy exploded to reach a level of 18.3/100.000 inhabitants per year in 2010. In the same hospital, proportion of Cesarean section among women delivering at hospital reached 52.2% (with 15% of expected deliveries taking place at hospital). Key-informant interpretation of the underlying factors explaining that increase in the number of major interventions in Bunia was the recruitment of a surgeon, the closure of the MSF hospital, and the PBF (Figure 4).

Interpretation of the relationship between observed events and production trends of the two hospitals, with a focus on agent interactions. Agents’ behavior has been different in Bunia and Logo hospitals, in the face of two PS9FED interventions, i.e. the development of a hospital plan and the PBF. In the Bunia hospital, the hospital staff (HS) reported being uninvolved and unsupported by the management team (MT) in the conception of the hospital development plan (KI7, KI10), to which they were not adapted (KI1). This plan did not have a significant impact (KI4). The management team (MT) reported not being supervised or supported by the provincial health division (KI1), which was represented by the hospital owner (HO) during the implementation of the hospital plan. The PBF, introduced by PS9FED, showed at one point a delay in the payment of fees in all the HD (KI1). Despite reasons given by the PS9FED technical assistants and sensitization by the MT, the HS went on strike (KI4). At the Logo hospital, the hospital staff adapted to the hospital plan and reported having been involved in its development (KI1, KI2). Despite the delay in payments from PS9FED (KI1, KI2, KI3, KI5, KI7), the hospital staff did not go on strike. The communication with the management team was considered to be good. The management team expressed appreciation for PS9FED technical assistants (KI1, KI2). The way the change of key actors influences the system is also interesting. The replacement of the district medical officer and the hospital director did not disrupt much the hospital management in Logo. Indeed, it has been the use in that hospital to always have another medical doctor ready to take over DMO or hospital director role. This strategy was perceived by all interviewed key-informants as being very positive. The replacement of the leader in charge of the diocesan medical works for the Mahagi diocese was, on the one hand, seen as a huge loss for the hospital (KI1, KI2, KI3, KI7, KI8), and on the other and, seen as a liberation for the management team when it comes to making management decisions (KI4, KI9). As a summary, our interpretation of the characteristics of interactions between agents is that in Bunia hospital, the cohesion between management team, hospital staff and hospital owners is much weaker than in Logo hospital.

## Discussion

This article aimed to identify, through an understanding of characteristics of the interactions between key agents, the necessary conditions for a hospital to be effective and capable of adapting to the changing environment. Two key issues emerged from the results: (1) the limits of using services productions as an indication of successful hospital development; (2) the interest of looking at hospital as a complex adaptive system to understand its governance. The limits of using services production as an indication of good hospital performances. Yet too often, success of an intervention (such as hospital plan or PBF) or a program is measured through “output” increase. In our study, intervention or program may appear more successful in Bunia than Logo. However, a closer look at the production of services raises some suspicion on the usefulness of some of the services provided. As such, the proportion of Cesarean section in Bunia (more than 50% of hospital deliveries in 2010) and the sharp increase of appendectomy raise questions on their indication. As a result, the increase of some services, rather than being an indicator of good hospital performance, may increase the risk of iatrogenic effect on the population. This has already been mentioned as a potential negative effect of PBF: paying for output may result in opportunistic behavior of providers who will seek to maximize their profit, even if it is at the expense of population health [17].

Looking at the hospital “working atmosphere", situation in Logo appears much better than in Bunia, even if services production is relatively stable over time. In that hospital, the perceived benefit of hospital development plan and the “resilience” to tensions resulting from delayed payment in the PBF implementation contrast clearly with the situation of Bunia. Indeed, no union strikes, better appropriation of the hospital plan, more hospital staff involvement, good collaboration between the hospital owner and the management team were observed on Logo. This difference may be better analyzed by looking at both hospitals as CAS. The interest of looking at hospital as a complex adaptive system to understand hospital governance. Making reference to the governance definition given by Rijke (i.e. actors network, institutional framework and process) [8], the use of CAS as a lens allowed us to interpret how an actors’ network may have remained stable or changed as a result of an institutional framework and may explain the production of hospital services. As such this made us moving beyond simplistic question about PBF or hospital plan effectiveness. The modeling of HGR actors’ network behavior through interactions between 3 key agents (the management team (MT), the hospital staff (HS) and hospital owners (HO)) identified the following possible patterns of process: (a) dynamic and coherent interaction between agents (this is the case in Logo) (b) dynamic and incoherent interaction between all agents (this is the case in Bunia). In the first pattern (the situation of Logo Hospital), a seemingly stagnating situation in health services production may be interpreted as desirable equilibrium in the actors’ network behavior. In the face of a changing environment (armed conflict with the arrival of a new program, for example), application of an institutional culture and adhesion to hospital objectives by all agents makes interaction between the 3 key-agents very strong. Their behavior is coherent one with the other. It helps the hospital to better adapt to this changing situation. In the second pattern (the situation of Bunia hospital), the influence of the context appears much more important. Closure of close-by hospital combined with development program interventions (PBF and hospital plan) is associated with important changes in hospital production. At first glance, this appears as a sign of program effectiveness. However, a closer analysis shows that some of the services (i.e. Cesarian Section) are probably over-produced, showing a lack of internal regulation of providers behavior. Indeed, in this case, interactions between the 3 key agents making the actors’ network, are either non- existing, either conflictive (see the strike of health personnel). This leads to over-reaction of the hospital to the context. These two patterns and the 2 hospitals’ behavior in reaction to the development program are illustrative of two key characteristics of CAS.

On the one hand the path dependency is important in understanding the institutional framework [13]. Indeed, both hospital adaptations depend heavily on pre-existing institutional arrangements and their evolution over time. Actions and reactions of each agent contribute to the achievement of shared institutional objectives and positively influence the production of care in Logo hospital [18]. On the contrary, we may hypothesize that in Bunia ineffective command-and-control mechanisms, let agents free to develop behavior independently from any institutional arrangement. In that situation, the dynamic interaction observed between agents is therefore incoherent (strikes, non-appropriation of the hospital plan) and opportunistic. On the other hand, the radical changes in production of services in Bunia could be interpreted as a phase transition. However, that change in services production could rather be the symptom of a heavily unstable system, highly influenced by contextual factor (in this case, PS9FED and closure of a nearby hospital). It is unfortunately not possible to asses if after that “phase transition", a new equilibrium was achieved or if production varied up and down. In evaluating PS9FED, the use of a case study as the research method helped to bring more in-depth understanding about the specific reality over time of two hospitals. We validate as much as possible the veracity of the data through triangulation between documents and key informants’ interview. However, we are aware that some perspective is absent. Namely, health beneficiaries were not included in the pool of informants. The results generated by this study do not aim to be generalized. Rather, the approach and questions raised in the discussion section, as a result of data analysis, are probably the main issues that would benefit external readers. Indeed, the two hospitals chosen were amongst those considered as good hospitals in Ituri (they are not representative of all that province). Also, the groups of informants interviewed were heavily involved in the implementation of the program.

## Conclusion

By studying 2 HGR through the lens of a CAS, we investigated how two dimensions of governance (actors’ network and institutional framework) evolved over time. In that perspective, aid effectiveness takes a different meaning. Performance is not only measured through increased hospital production (as may be often the case in development aid evaluation) but through meaningful process of hospital actors’ network adaptation. Expected process is not necessarily a change; strengthened equilibrium may be a preferable result. Much more attention should be given in future international aid to the proper understanding of the hospital governance and the way to strengthen it.
